# Interferon alpha treatment stimulates interferon gamma expression in type I NKT cells and enhances their antiviral effect against hepatitis C virus

**DOI:** 10.1371/journal.pone.0172412

**Published:** 2017-03-02

**Authors:** Eisuke Miyaki, Nobuhiko Hiraga, Michio Imamura, Takuro Uchida, Hiromi Kan, Masataka Tsuge, Hiromi Abe-Chayama, C. Nelson Hayes, Grace Naswa Makokha, Masahiro Serikawa, Hiroshi Aikata, Hidenori Ochi, Yuji Ishida, Chise Tateno, Hideki Ohdan, Kazuaki Chayama

**Affiliations:** 1 Department of Gastroenterology and Metabolism, Applied Life Science, Institute of Biomedical & Health Science, Hiroshima University, Hiroshima, Japan; 2 Liver Research Project Center, Hiroshima University, Hiroshima, Japan; 3 Laboratory for Digestive Diseases, Center for Genomic Medicine, The Institute of Physical and Chemical Research (RIKEN), Hiroshima, Japan; 4 PhoenixBio Co., Ltd., Higashihiroshima, Japan; 5 Department of Surgery, Division of Frontier Medical Science, Programs for Biomedical Research, Graduate School of Biomedical Science, Hiroshima University, Hiroshima, Japan; Saint Louis University, UNITED STATES

## Abstract

Interferon (IFN) inhibits hepatitis C virus (HCV) replication through up-regulation of intrahepatic IFN-stimulated gene expression but also through activation of host immune cells. In the present study, we analyzed the immune cell-mediated antiviral effects of IFN-α using HCV-infected mice. Urokinase-type plasminogen activator (uPA)-severe combined immunodeficiency (SCID) mice with transplanted human hepatocytes were infected with genotype 1b HCV and injected with human peripheral blood mononuclear cells (PBMCs). IFN-α treatment following human PBMC transplantation resulted in a significant reduction in serum HCV RNA titers and a higher human CD45-positive mononuclear cell chimerism compared to mice without human PBMC transplantation. In mice with human PBMCs treated with IFN-α, serum concentrations of IFN-γ increased, and natural killer T (NKT) cells, especially type I NKT cells, produced IFN-γ. Mice in which IFN-γ signaling was blocked using antibody or in which transplanted PBMCs were depleted for type I NKT cells showed similar levels of anti-HCV effect compared with mice treated only with IFN-α. These results show that IFN-α stimulates IFN-γ expression in type 1 NKT cells and enhances the inhibition of HCV replication. We propose that type 1 NKT cells might represent a new therapeutic target for chronic hepatitis C patients.

## Introduction

Approximately 170 million people worldwide are chronically infected with hepatitis C virus (HCV) [1]. As a major risk factor for cirrhosis and hepatocellular carcinoma (HCC), HCV infection is one of the leading causes of cancer-related deaths [2]. The current therapy for HCV is the combination of two or more direct-acting antivirals (DAAs) without interferon (IFN) [3, 4]. Although these therapies are able to achieve a high sustained virological response (SVR) rate, there are a few patients who nonetheless fail to respond to the treatment. The most important factor is the existence of resistance-associated variants (RAVs) to one or more of the DAAs. IFN is a treatment option for chronic hepatitis C patients with DAA RAVs.

IFN eliminates HCV through both direct and indirect effects on hepatocytes [5]. IFN directly binds to HCV-infected hepatocyte surface receptors and inhibits HCV replication by inducing the expression of IFN-stimulated genes (ISGs) [6]. Indirectly, IFN activates host immune cells, such as natural killer (NK) cells and T cells [7], and these activated immune cells exhibit antiviral activity by producing cytokines. NK cells were reported to produce cytokines and suppress HCV replication following activation by IFN [8–13]. T cells have also been reported to suppress HCV activity after stimulation by IFN [7, 14–16].

Natural killer T (NKT) cells are a unique subset of lymphocytes that co-express T-cell receptors and NK cell markers [17]. NKT cell activation was reported to be correlated with subsequent T cell response [18], resulting in depletion of chronic intrahepatic NKT cell populations and reduction of HCV infection [19]. However, little is known about the role of NKT cells in HCV infection.

We previously reported an animal model of HCV-infected human hepatocyte transplanted chimeric mice by transplanting human liver lymphocytes using urokinase-type plasminogen activator-severe combined immunodeficiency (uPA-SCID) mice [20]. In this study, we investigated the IFN-induced immune response using the same mouse model with HCV-infection and human immunity by transplanting human peripheral mononuclear cells (PBMCs).

## Materials and methods

### Generation of human hepatocyte chimeric mice

Generation of the uPA^+/+^/SCID^+/+^ mice and transplantation of human hepatocytes with HLA-A24 were performed as described previously [21]. All mice were transplanted with frozen human hepatocytes obtained from the same donor. Mouse serum concentrations of human serum albumin (HSA), which is correlated with the liver repopulation index, were measured as described previously [21]. All animal protocols described in this study were performed in accordance with the Guide for the Care and Use of Laboratory Animals and the local committee for animal experiments, and the experimental protocol was approved by the Ethics Review Committee for Animal Experimentation of the Graduate School of Biomedical Sciences, Hiroshima University.

### Human serum samples

Human serum samples containing high titers of genotype 1b HCV RNA (2.2 x 10^6^ copies/mL) were obtained from patients with chronic hepatitis who provided written informed consent. Individual serum samples were divided into aliquots and stored in liquid nitrogen. Six weeks after hepatocyte transplantation, chimeric mice were injected intravenously with 50 μL of HCV-positive human serum.

### Preparation of human PBMCs and transplantation into human hepatocyte chimeric mice

PBMCs were isolated using Ficoll-Hypaque density gradient centrifugation from a healthy blood donor with HLA-A24. Eight to ten weeks after HCV inoculation, 4×10^7^ human PBMCs were transplanted into human hepatocyte chimeric mice. To assess the effect of depletion of human type I NKT cells from administered PBMCs on hepatitis formation, Anti-iNKT Micro Beads (Milteny Biotec, CA, USA) were used.

### Quantitation of HCV RNA and ISG mRNAs

RNA was extracted from mouse serum and liver samples by SepaGene RV-R (EIDIA Co.,LTD., Tokyo, Japan), and dissolved in 8.8 μL RNase-free water. Extracted RNA was reverse transcribed using random primer (Takara Bio Inc., Shiga, Japan) and M-MLV reverse transcriptase (ReverTra Ace, TOYOBO Co.,LTD., Osaka, Japan) in 20 μL reaction mixture according to the instructions provided by the manufacturer. Nested polymerase chain reaction (PCR) and HCV quantitation by Light Cycler (Roche Diagnostics K.K., Tokyo, Japan) were performed as previously described [22]. Quantitation of ISG expression (*HLA-DMB*, GTP-binding protein [*GBP5*]) was performed using real-time PCR Master Mix (TOYOBO) and TaqMan Gene Expression Assay primer and probe sets (PE Applied Biosystems, Foster City, CA). Thermal cycling conditions were as follows: a precycling period of 1 min at 95°C followed by 40 cycles of denaturation at 95°C for 15 s and annealing/extension at 60°C for 1 min. Expression levels of each ISG are expressed as a ratio with respect to β-actin levels.

### Treatment of mice with IFN and anti-IFN-γ antibody

To analyze the immune response to IFN, mice received daily intramuscular injections of either 1000 IU/g of human IFN-α (Dainippon Sumitomo Pharma Co., Tokyo, Japan) or human IFN-γ (Shionogi & Co., Ltd, Osaka, Japan) for 7 days after human PBMC transplantation. To analyze the antiviral effect of IFN-γ, mice received intraperitoneal injections with either 1.5 mg of anti-human IFN-γ antibody or isotype antibody (R&D Systems, Minneapolis, MN) one day before human PBMC transplantation. Anti-human IFN-gamma antibody has no cross-reactivity with mouse IFN-gamma, and blocks human IFN-gamma specifically.

### Flow cytometry

We collected mouse liver infiltrating cells flowing through the portal vein after hepatectomy [23]. Reconstructed human PBMCs in mice were analyzed by flow cytometry with the following mAbs used for surface and intracellular staining: APC-H7 Anti-Human cluster of differentiation (CD)3 (clone SK7), APC conjugated anti-CD4 (clone SK), BD Horizon™ V450 Anti-Human CD8 (clone RPA-T8), HU HRZN V500 MAB conjugated anti-Human CD45 (clone H130), Alexa Fluor 488 conjugated anti-Human CD56 (clone B159), PE conjugated anti-Human CD25 (clone M-A251), and PE-Cy7-conjugated anti-Mouse CD45 (clone 30-F11). Each of the above mAbs was purchased from BD Bioscience. PE-conjugated IFN-γ (clone 45.15), FITC-conjugated TCRα24 (clone C15) and APC-conjugated TCRβ11 (clone C21) were purchased from Beckman Coulter Co., (Tokyo, Japan). Dead cells identified by light scatter and propidium iodide staining were excluded from the analysis. For intracellular staining, cells were permeabilized and fixed after surface staining using the BD Cytofix/Cytoperm kit (BD Bioscience, Heidelberg, Germany). Flow cytometry was performed using a FACSAria™ II flow cytometer (BD Bioscience), and results were analyzed with FlowJo (TreeStar). NKT cells were defined as human CD3^+^CD56^+^ cells, and type I NKT cells were defined as Vα24^+^ and Vβ11^+^ cells.

### Histochemical analysis of mouse liver

Histochemical analysis and immunohistochemical staining using antibodies against HSA (Bethyl Laboratories Inc., Montgomery, TX) were performed as described previously [24–26]. Immunoreactive materials were visualized using a streptavidin-biotin staining kit (Histofine SABPO kit; Nichirei, Tokyo, Japan) and diainobenzidine.

### Cytokine assay

The concentrations of human IFN-γ, granzyme A, granzyme B, interleukin (IL)-2, IL-4, IL-6, IL-8, IL-10, IL-12p70, IL-13, IL-17A and mouse IFN-γ were quantified by chemokine cytometric bead array (CBA) kits (BD Biosciences, Heidelberg, Germany) in accordance with the manufacturer’s instructions. The minimum and maximum detection limits were 10 and 2500 pg/mL, respectively. Mouse serum cytokine levels were measured before and 4 and 7 days after IFN-α injection.

### ALT measurements

Human alanine aminotransferase (ALT) levels in mouse sera were measured using Fuji DRI-CHEM (Fuji Film, Tokyo, Japan) according to the manufacturer’s instructions.

### Statistical analysis

Changes in mouse serum HSA, HCV RNA levels, the frequencies of human PBMCs in mouse livers, expression of ISGs and serum ALT levels were compared by Mann-Whitney U and unpaired t-tests. *P* values less than 0.05 were considered statistically significant.

## Results

### Human PBMCs enhanced the antiviral effects of IFN-α in HCV-infected human hepatocyte chimeric mice

**Fig 1:**
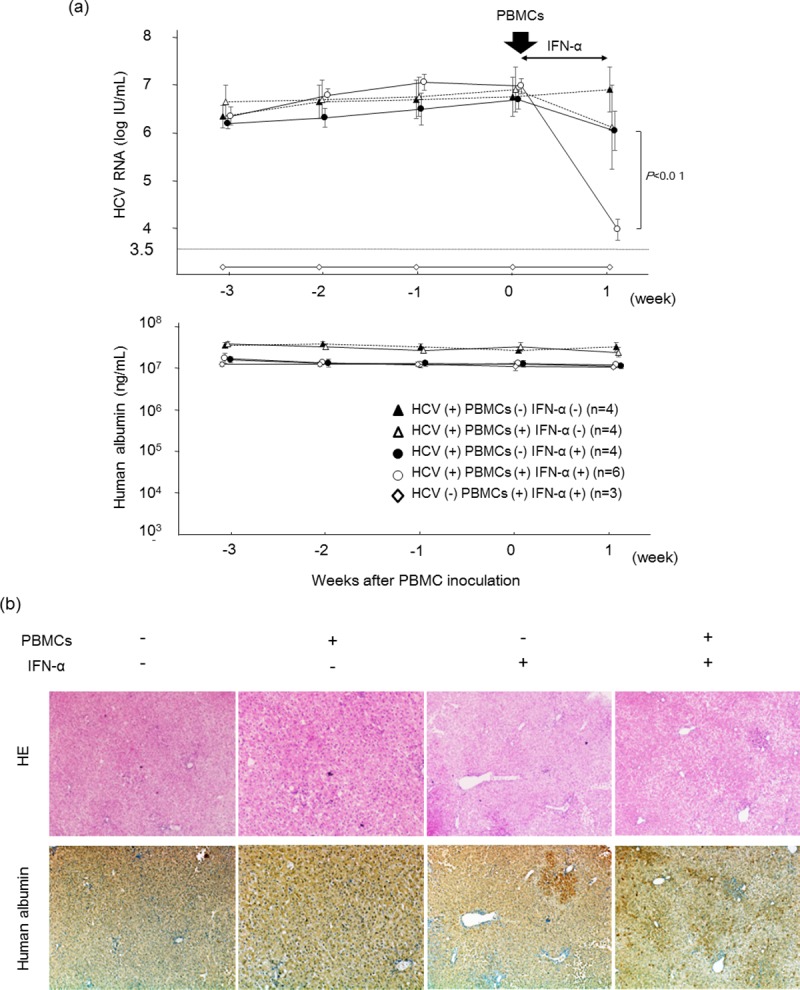
Treatment with human PBMCs and IFN-α in HCV-infected human hepatocyte chimeric mice. HCV-infected human hepatocyte chimeric mice were injected with or without 4×10^7^ human PBMCs, and then treated with or without 1000 IU/kg of IFN-α for seven days. (a) Time course of HCV RNA titer (upper panel) and human albumin (lower panel) in mouse serum. Data are presented as the mean ± SD. Mice without HCV infection were also shown. (b) Histological analysis of liver samples obtained from mice. Liver samples at seven days after injection of human PBMCs were stained with hematoxylin-eosin staining (HE) and immunohistostained with anti-HSA antibody (human albumin). Most cells in the regions shown are human hepatocytes (original magnification, ×40).

HCV-infected human hepatocyte chimeric mice were treated with 4×10^7^ human PBMCs obtained from a healthy volunteer. PBMC treatment showed a slight reduction in mouse serum HCV RNA levels (Fig 1A). Seven days of human IFN-α treatment without human PBMC injection in mice reduced serum HCV RNA levels by 1.23 ± 0.15 log, whereas in combination with PBMCs, HCV RNA levels were reduced by 2.5 ± 0.45 log (*P*<0.01). Mouse serum HSA levels did not decrease during these treatments. Histological examination showed that neither human PBMC injection nor treatment with IFN-α resulted in apparent damage to human hepatocytes at seven days after treatment (Fig 1B). Serum human ALT levels in mouse serum also showed no increase in PBMC- or IFN-α-treated mice (S1 Fig). Therefore, the reduction of HCV in mice with PBMC injection and IFN-α treatment was concluded not to be due to toxicity of these treatments.

### Analysis of liver-infiltrating human lymphocytes in mice

**Fig 2:**
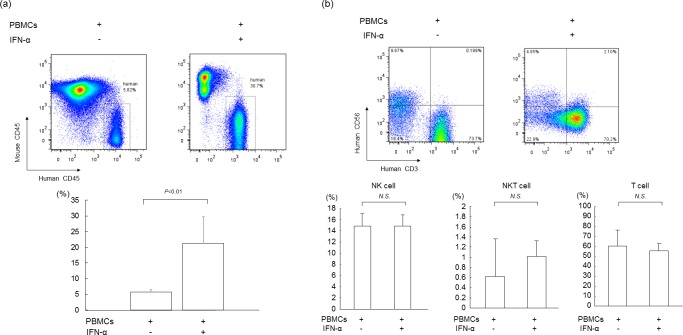
Analysis of liver infiltrating human lymphocytes in mice with human PBMCs. HCV-infected human hepatocyte chimeric mice were injected with 4×10^7^ human PBMCs, then treated with (n = 6) or without (n = 4) 1000 IU/kg of IFN-α for seven days. Liver mononuclear cells were isolated from mice seven days after human PBMC treatment. (a) Liver mononuclear cells were stained with antibodies against human CD45 and mouse CD45, and analyzed by flow cytometry (upper panel). Statistical analysis of differences in the percentage of human mononuclear cells in PBMCs-treated mice with or without IFN-α is shown in the lower panel. Data are presented as the mean ± SD. (b) Liver mononuclear cells were stained with antibodies against human CD3 and human CD56 and analyzed by flow cytometry (upper panel). Percentages of human mononuclear cells in PBMCs-treated mice are shown in the lower panel. Data are presented as the mean ± SD.

We analyzed liver-infiltrating cells for human cell surface markers in mice injected with human PBMCs. Flow cytometry analysis showed that treatment with 7 days of IFN-α resulted in significantly greater human CD45-positive mononuclear cell chimerism in HCV-infected mice compared to mice without IFN-α treatment (21.2 ± 8.5% and 5.8 ± 0.8%, respectively; *P*<0.01) (Fig 2A). In IFN-α-treated mouse livers, the frequencies of NK, NKT and T cells were not changed, indicating that the numbers of each of these cells increased following IFN-α treatment (Fig 2B).

### IFN-γ is essential for antiviral effect in mice treated with human PBMCs and IFN-α

**Fig 3:**
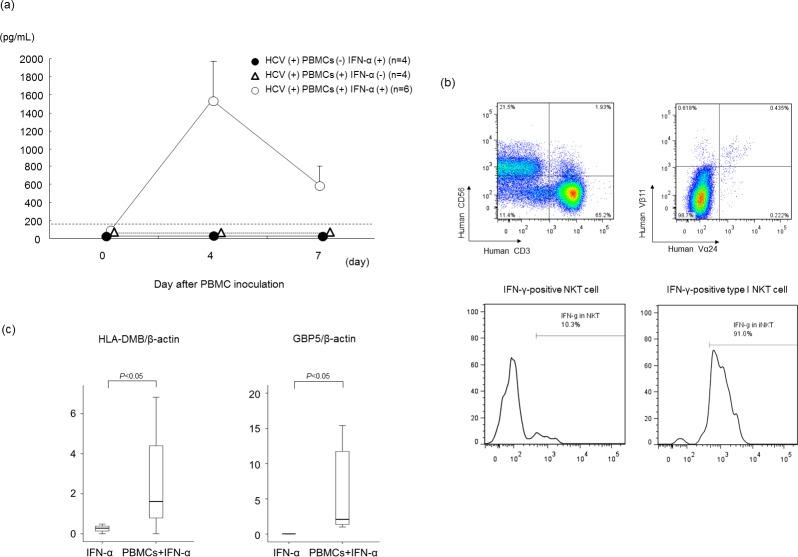
Up-regulated IFN-γ inhibits HCV replication in mice treated with human PBMCs and IFN-α. Mice were treated as described in the legend to Fig 2. (a) Time course of serum IFN-γ concentration in mice treated with or without IFN-α. Mice treated with IFN-α without PBMC injection were also analyzed. Data are presented as the mean ***±*** SD. (b) Liver mononuclear cells were stained with antibodies against human CD3 and CD56, human TCR Vα24, and TCR Vβ11 and analyzed by flow cytometry (upper panel). The frequency of IFN-γ-positive cells in NKT cells, and type I NKT cells were analyzed (lower panel). (c) Twenty-four hrs after IFN-α injection, intrahepatic gene expression levels of *HLA-DMB* and GTP-binding protein 5 (*GBP5*) were measured. RNA levels are expressed relative to β-actin mRNA. Data are represented as the mean ± SD of 3 mice.

The cytokine time course assay demonstrated that mouse serum human IFN-γ increased four days after human PBMC injection and IFN-α treatment (Fig 3A). No up-regulation of human IFN-γ was observed in either PBMC- nor IFN-α-treated mice. Other cytokines such as TNF-α (Th1), IL-2 (Th1), IL-4 (Th2), IL-17 (Th17) were undetectable. Flow cytometry analysis showed that NKT cells, especially type I NKT cells, produced IFN-γ at seven days after treatment (Fig 3B). Although the populations of type I NKT cells did not increase, the number of IFN-γ producing type I NKT cells increased dramatically following IFN-α treatment (S2 Fig). These IFN-γ-producing type I NKT cells were more abundant compared to T cells or NK cells (S3 Fig). Along with up-regulation of IFN-γ, the expression of intrahepatic IFN-γ-specific ISGs such as *HLA-DMB* and *GBP5* had increased in human PBMC- and IFN-α-treated mice 24 hrs after IFN-α injection (Fig 3C), suggesting that these ISGs contribute to serum HCV RNA reduction following IFN-α treatment.

**Fig 4:**
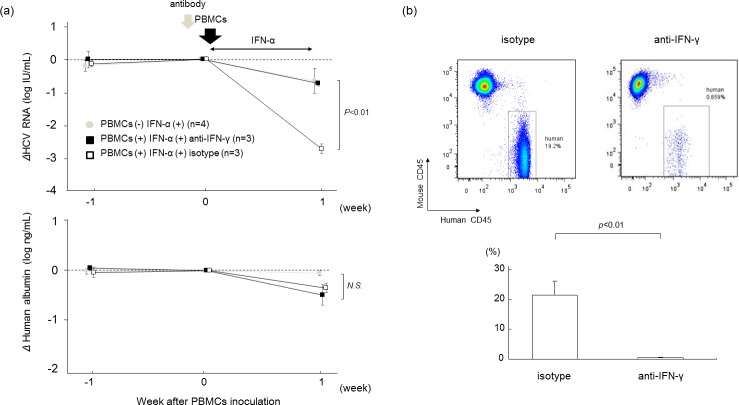
Effect of antibodies against IFN-γ. HCV-infected human hepatocyte chimeric mice were injected with 4×10^7^ human PBMCs. The mice were then treated with 1000 IU/kg of IFN-α for 7 days. One day before human PBMC transplantation, mice were injected either with isotype (n = 3) or with antibody against IFN-γ (n = 3). (a) Reductions of serum HCV RNA levels and human albumin concentrations are shown. Mice treated with IFN without PBMCs injection were also analyzed (gray circles). Data are presented as the mean ± SD. (b) Liver mononuclear cells isolated from mice were stained with antibodies against human CD45 and mouse CD45, and analyzed by flow cytometry (upper panel). Statistical analysis of the frequencies of human mononuclear cells in PBMC-treated mice are shown in the lower panel. Data are presented as the mean ± SD.

To assess the role of IFN-γ in HCV inhibition, a blocking monoclonal antibody (mAb) against human IFN-γ was administered to mice before addition of human PBMCs and IFN-α treatment. Treatment of mice with anti-IFN-γ antibody before IFN-α treatment eliminated the antiviral effect of human PBMCs and IFN-α (Fig 4A). Development of human CD45-positive mononuclear cell chimerism by treatment with human PBMCs and IFN-α treatment was also completely negated by anti-IFN-γ antibody (Fig 4B), suggesting that IFN-γ produced by NKT cells is essential not only for its direct antiviral effect, but also for the development of human lymphocyte chimerism in mice treated with human PBMCs and IFN-α.

### Effect of Type I NKT cell depletion on antiviral effect

**Fig 5:**
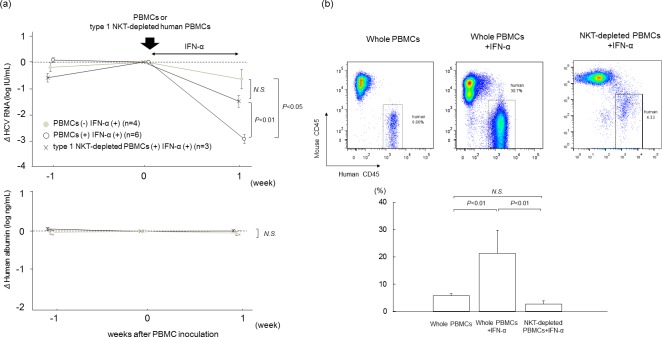
Treatment with human PBMCs with depletion of type I NKT cells. HCV-infected human hepatocyte chimeric mice were injected with whole human PBMCs or type 1 NKT-depleted human PBMCs (n = 3), and then treated with (n = 6) or without (n = 4) 1000 IU/kg of IFN-α for seven days. (a) Reduction of serum HCV RNA levels (upper panel) and human albumin concentrations (lower panel) are shown. IFN-α treated mice without human PBMC transplantation were also analyzed. Data are presented as the mean ± SD. (b) Liver mononuclear cells isolated from mice seven days after human PBMC treatment were stained with antibodies against human CD45 and mouse CD45, and analyzed by flow cytometry (upper panel). Statistical analysis of the frequencies of human mononuclear cells in PBMCs-treated mice is shown in the lower panel. Data are presented as the mean ± SD.

To confirm the necessity of type I NKT cells for antiviral effect, we depleted type I NKT cells with negative selection using antibody-coated magnetic beads before administration of PBMCs. Serum HCV RNA levels in mice treated with type I NKT-depleted PBMCs and IFN-α decreased to levels similar to that of mice treated only with IFN-α (Fig 5A). Development of human CD45-positive mononuclear cell chimerism was also prevented in mice administered with type I NKT-depleted PBMCs and IFN-α, and the chimerism decreased to the same level as in mice without IFN-α treatment (Fig 5B).

## Discussion

NKT cells are thought to serve as a bridge between innate immune cells, including NK cells and dendritic cells, and acquired immune effectors, consisting of T cells [27]. The network of activation initiated by NKT cells extends to activation of B cells and T cells. We previously reported that although human PBMCs in human hepatocyte chimeric mice were detected during the first week, those cells were almost undetectable by day 14 because of the activity of mouse NK cells and macrophages [24]. It was also thought that IFN-induced acute graft-versus-host disease occurred about twenty days after the transplantation [28]. Therefore, we analyzed the antiviral effect at seven days after the injection of human PMBCs.

In the present study, mice serum HCV RNA levels decreased by approximately 3 log IU/mL after PBMC transplantation and treatment with human IFN-α for seven days. In patients with chronic hepatitis C, one week of daily IFN-α treatment decreased serum HCV RNA levels by 2–3 log IU/mL [29]. Viral kinetics following IFN-α treatment in our mouse model appears to be similar with that of chronic hepatitis C patients. The reduction of serum HCV RNA levels in PBMC-transplanted mice treated with IFN-α was significantly higher than in mice without PBMC transplantation (Fig 1A). Based on the lack of increase in ALT levels (S1 Fig), unchanged human albumin levels (Fig 1A) and no apparent change in liver histology (Fig 1B), it was concluded that no liver damage was inflicted by treatment with human PBMCs and IFN. Based on our results, we hypothesized that an indirect effect of IFN in our mouse model was to enhance antiviral activity without damaging human hepatocytes. First, we analyzed the liver infiltrating human lymphocytes with flow cytometry. Surprisingly, there was a significantly higher human CD45-positive mononuclear cell chimerism in PBMC-transplanted mice that were treated with 1000 IU/g of IFN-α than in other mice (Fig 2A). Second, we examined the time course of serum concentrations of several cytokines in mouse serum. Although the concentration of cytokines was below the detection limit in most mice, administration of IFN-α significantly increased serum concentration of human IFN-γ four days after PBMC injection (Fig 3A). Third, we analyzed the infiltration of mononuclear cells in mouse liver with flow cytometry to identify IFN-producing cells, including NK cells, T cells and NKT cells [30]. We detected IFN-γ-positive cells among the NKT cells. NKT cells are divided into type I and type II subsets [31]. Type I NKT cells are pro-inflammatory and secrete IFN-γ and are defined by a restricted T-cell receptor repertoire, which in humans consists of the T-cell receptor (TCR) chains Vα24-Ja18 and Vβ11 with a conserved CDR3 [32]. In this study, the majority of type I NKT cells of HCV-infected mice treated with IFN-α was IFN-γ-positive (Fig 3B). IFN-γ-specific ISGs [33] were significantly more strongly up-regulated in PBMC-transplanted chimeric mice treated with IFN-α than in mice without human PBMCs (Fig 3C).

We performed several experiments to assess the importance of IFN-γ and verify the presence of NKT cells. The inhibition of IFN-γ by anti-IFN-γ antibody dramatically reduced the antiviral effect (Fig 4A) and significantly decreased human CD45-positive cell chimerism in HCV-infected mice (Fig 4B). Although levels of human IFN-γ in mouse serum increased following administration of human PBMCs and IFN-α, mice IFN-γ levels were below the detectable limit (data not shown). These results showed that the effect of IFN-γ antibody was due to the inhibition of human IFN-γ produced from human PBMCs, and IFN-γ was crucial to the immune response. The antiviral effect of HCV-infected mice injected with type I NKT cell-depleted PBMCs was similar to the effect in HCV-infected mice treated only with IFN-α (Fig 5A), and human CD45-positive cell chimerism was similar to that in HCV-infected mice without IFN-α (Fig 5B).

To determine the antiviral effect of exogenous IFN-γ, we administered human IFN-γ to HCV-infected mice daily for one week (S4 Fig). In contrast to IFN-α, IFN-γ treatment showed no antiviral effect, indicating that local IFN-γ secreted from NKT cells in the livers seems to play an important role in antiviral activity in this mouse model.

NKT cells usually account for between 0.001–3% of human peripheral blood mononuclear cells [34]. In this study, the proportion of NKT cells was around 1% of human CD45-positive cells (Fig 2B). Despite this low frequency, depletion of human type I NKT cells from administered PBMCs decreased the anti-viral effect of IFN-α, indicating that type I NKT cells seem to play an important role on IFN-α-induced anti-HCV activity.

In summary, we analyzed immune cells present after treatment with IFN-α in HCV-infected human hepatocyte chimeric mice transplanted with human PBMCs. The IFN-γ secreted from NKT cells activated other immune cells and increased the antiviral effect. NKT cell-targeted adjuvant cell therapies were initially developed in clinical trials on cancer patients [27]. These NKT cells-targeted adjuvant cell therapies could be a new treatment option for refractory chronic hepatitis C.

## Supporting information

S1 Fig Changes in human alanine aminotransferase (ALT) levels in mouse serum. HCV-infected human hepatocyte chimeric mice were injected with 4×10^7^ human PBMCs, and then treated with or without 1000 IU/kg of IFN-α for seven days. Changes in human ALT levels in mouse serum are shown. Mice treated with PBMCs and IFN-α without HCV infection were also analyzed. Data are presented as the mean ± SD of 3 mice.
(TIF)

S2 Fig Analysis of the frequency of IFN-γ-positive cells in type I NKT. HCV-infected human hepatocyte chimeric mice were injected with 4×10^7^ human PBMCs, and then treated with (n = 6) or without (n = 4) 1000 IU/kg of IFN-α for seven days. Liver mononuclear cells were stained with antibodies against human TCR Vα24, and TCR Vβ11, and analyzed by flow cytometry. The frequency of type I NKT cells and IFN-γ-positive cells in type I NKT in PBMCs-injected mice are shown. Data are presented as the mean ± SD.
(TIF)

S3 Fig The frequency of IFN-γ-positive cells in T and NK cells. HCV-infected human hepatocyte chimeric mice were injected with 4×10^7^ human PBMCs, and then treated with 1000 IU/kg of IFN-α for seven days. Liver mononuclear cells were isolated from mice seven days after human PBMCs treatment. Liver mononuclear cells were stained with antibodies against human CD3 and CD56 and analyzed by flow cytometry. The frequency of IFN-γ-positive cells in T and NK cells were analyzed.
(TIF)

S4 Fig Treatment with IFN in HCV-infected human hepatocyte chimeric mice. HCV-infected human hepatocyte chimeric mice were injected with or without 4×10^7^ human PBMCs, and then treated with either 1000 IU/kg of FN-α or 20000 IU/kg of IFN-γfor 7 days. Reductions of serum HCV RNA levels and human albumin concentrations are shown. Data are presented as the mean ± SD.
(TIF)

## Abbreviations

ALT: alanine aminotransferase
DAA: direct acting antiviral
GBP: GTP-binding protein
HCV: hepatitis C virus
HSA: human serum albumin
IFN: interferon
ISGs: interferon-stimulated genes
NK: natural killer
PBMCs: peripheral blood mononuclear cells
uPA: urokinase-type plasminogen activator
SCID: severe combined immunodeficiency
SVR: sustained virological response
RAVs: resistance-associated variants
