# Supplementary figures and images for: Interferon alpha treatment stimulates interferon gamma expression in type I NKT cells and enhances their antiviral effect against hepatitis C virus
Source: PLoS One. 2017 Mar 2;12(3):e0172412. doi: 10.1371/journal.pone.0172412 (PMC5333814; doi:10.1371/journal.pone.0172412)

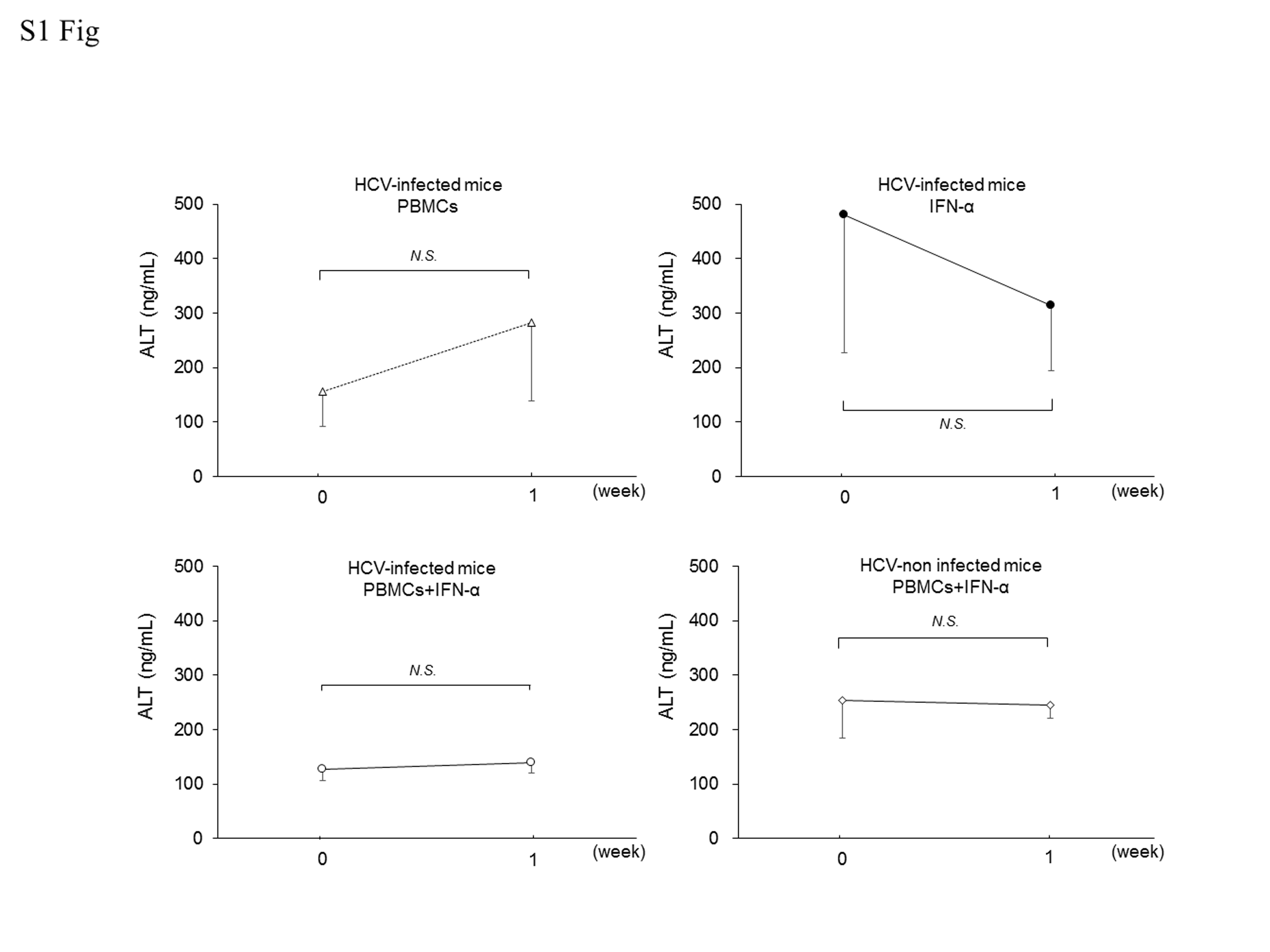

Supplement: S1 Fig — HCV-infected human hepatocyte chimeric mice were injected with 4×10^7^ human PBMCs, and then treated with or without 1000 IU/kg of IFN-α for seven days. Changes in human ALT levels in mouse serum are shown. Mice treated with PBMCs and IFN-α without HCV infection were also analyzed. Data are presented as the mean ± SD of 3 mice. (TIF) [file pone.0172412.s001.tif]

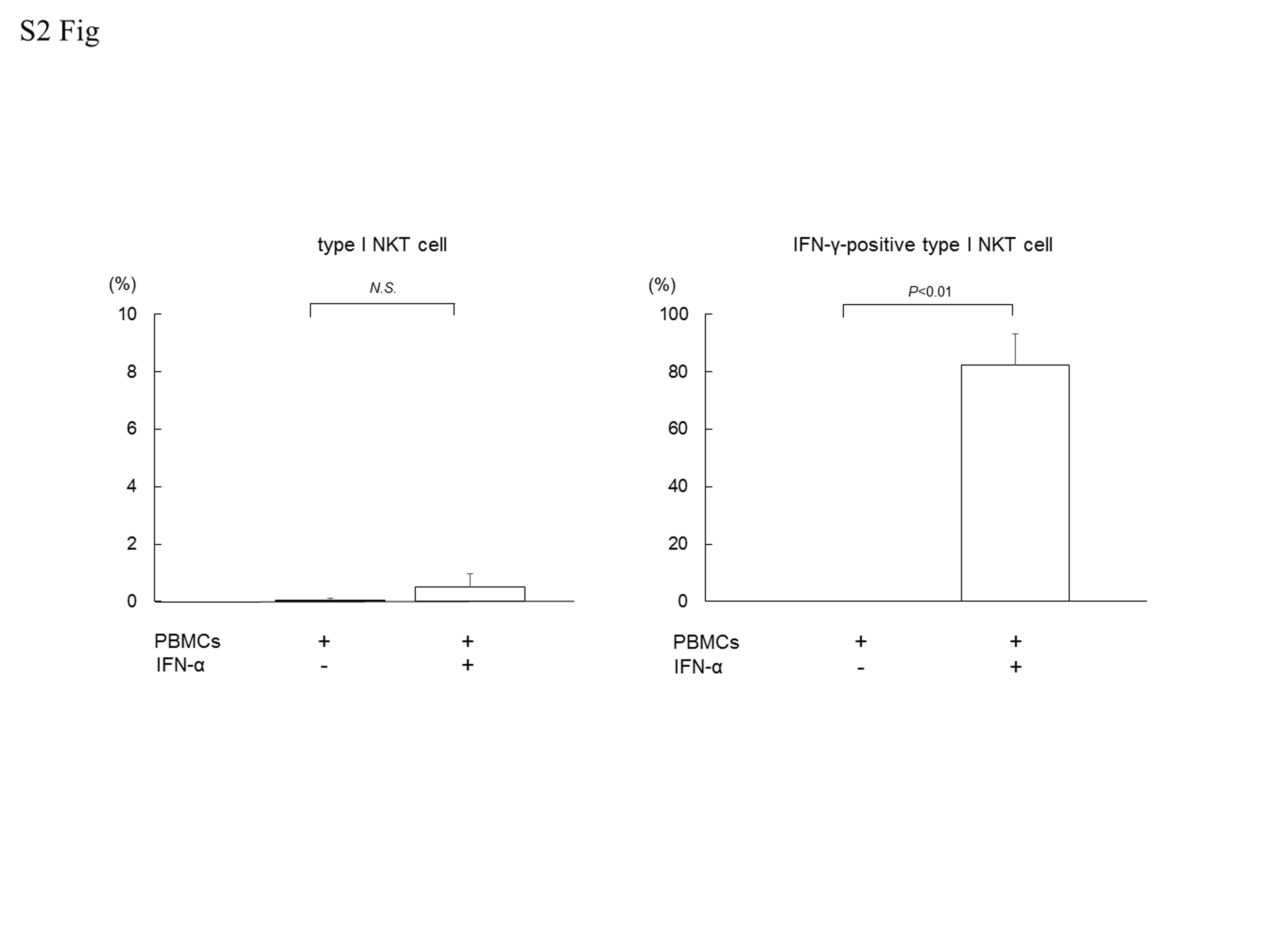

Supplement: S2 Fig — HCV-infected human hepatocyte chimeric mice were injected with 4×10^7^ human PBMCs, and then treated with (n = 6) or without (n = 4) 1000 IU/kg of IFN-α for seven days. Liver mononuclear cells were stained with antibodies against human TCR Vα24, and TCR Vβ11, and analyzed by flow cytometry. The frequency of type I NKT cells and IFN-γ-positive cells in type I NKT in PBMCs-injected mice are shown. Data are presented as the mean ± SD. (TIF) [file pone.0172412.s002.tif]

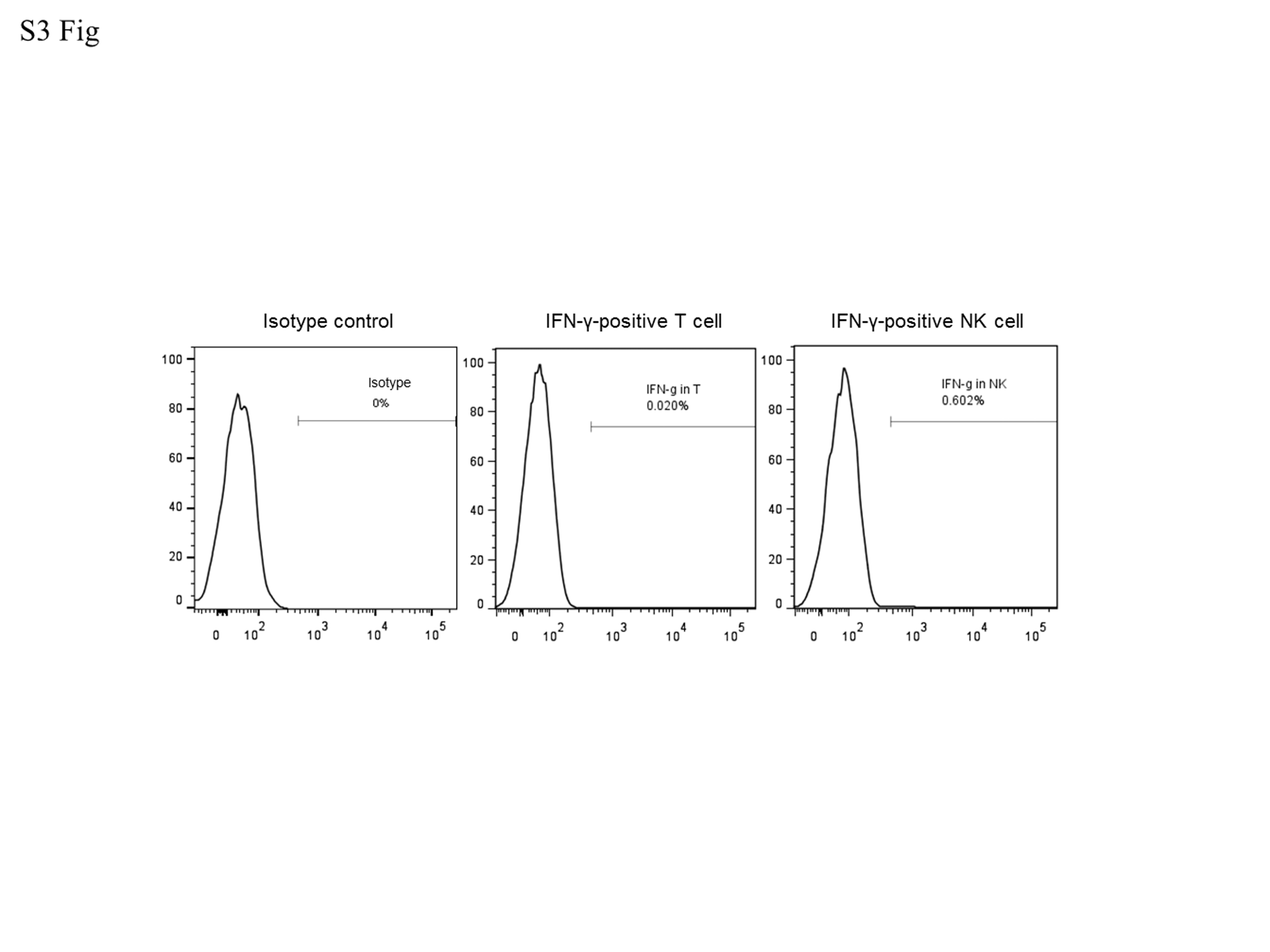

Supplement: S3 Fig — HCV-infected human hepatocyte chimeric mice were injected with 4×10^7^ human PBMCs, and then treated with 1000 IU/kg of IFN-α for seven days. Liver mononuclear cells were isolated from mice seven days after human PBMCs treatment. Liver mononuclear cells were stained with antibodies against human CD3 and CD56 and analyzed by flow cytometry. The frequency of IFN-γ-positive cells in T and NK cells were analyzed. (TIF) [file pone.0172412.s003.tif]

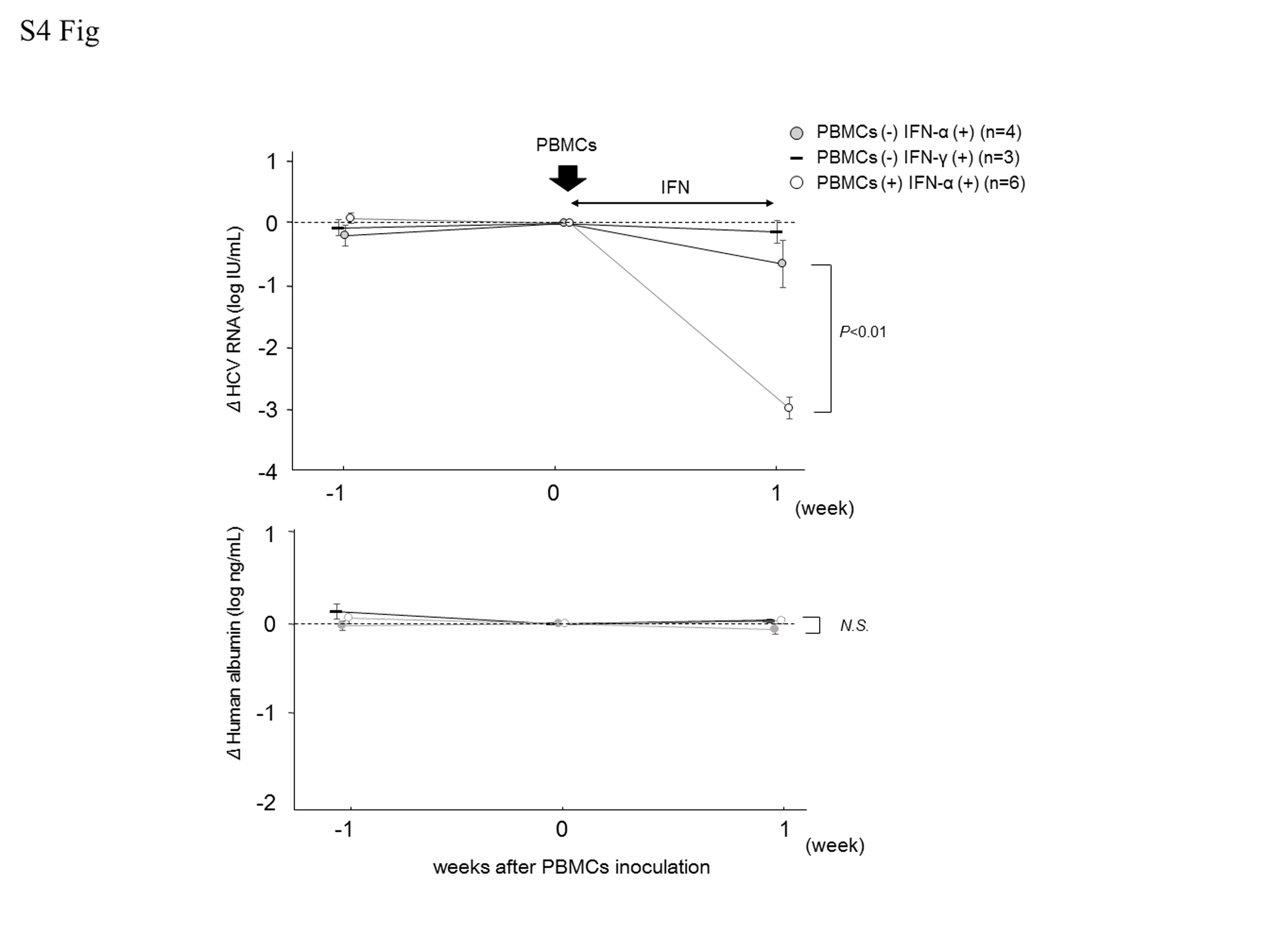

Supplement: S4 Fig — HCV-infected human hepatocyte chimeric mice were injected with or without 4×10^7^ human PBMCs, and then treated with either 1000 IU/kg of FN-α or 20000 IU/kg of IFN-γfor 7 days. Reductions of serum HCV RNA levels and human albumin concentrations are shown. Data are presented as the mean ± SD. (TIF) [file pone.0172412.s004.tif]
